# Personally Collected Health Data for Precision Medicine and Longitudinal Research

**DOI:** 10.3389/fmed.2019.00125

**Published:** 2019-06-04

**Authors:** Pierluigi D'Antrassi, Marco Prenassi, Lorenzo Rossi, Roberta Ferrucci, Sergio Barbieri, Alberto Priori, Sara Marceglia

**Affiliations:** ^1^Dipartimento di Elettronica, Informazione e Bioingegneria (DEIB), Politecnico di Milano, Milan, Italy; ^2^U.O. Neurofisiopatologia, Fondazione IRCCS Ca' Granda Ospedale Maggiore Policlinico, Milan, Italy; ^3^Dipartimento di Ingegneria e Architettura, Università degli Studi di Trieste, Trieste, Italy; ^4^Newronika S.r.l., Milan, Italy; ^5^Dipartimento di Scienze della Salute, “Aldo Ravelli” Research Center for Neurotechnology and Experimental Brain Therapeutics, Università degli Studi di Milano, Milan, Italy; ^6^UO Neurologia, ASST Santi Paolo e Carlo, Milan, Italy

**Keywords:** personal health records, personal health systems, personal health monitoring, telehealth, longitudinal studies

## Abstract

Health data autonomously collected by users are presently considered as largely beneficial for wellness, prevention, disease management, as well as clinical research, especially when longitudinal, chronic, home-based monitoring is needed. However, data quality and reliability are the main barriers to overcome, in order to exploit such potential. To this end, we designed, implemented, and tested a system to integrate patient-generated personally collected health data into the clinical research data workflow, using a standards-based architecture that ensures the fulfillment of the major requirements for digital data in clinical studies. The system was tested in a clinical investigation for the optimization of deep brain stimulation (DBS) therapy in patients with Parkinson's disease that required both the collection of patient-generated data and of clinical and neurophysiological data. The validation showed that the implemented system was able to provide a reliable solution for including the patient as direct digital data source, ensuring reliability, integrity, security, attributability, and auditability of data. These results suggest that personally collected health data can be used as a reliable data source in longitudinal clinical research, thus improving holistic patient's personal assessment and monitoring.

## Introduction

In the digital era, personal mHealth Apps combined with the Internet of Health Things (IoHT) technologies have the potential to help patients managing medical conditions, monitor lifestyles, and provide medical advice (1). Clinical research may benefit from such technologies as well: personally collected data may enable capturing the personal perspectives on new therapies compliance or tolerability, patient's conditions or symptoms. These new types of data, directly collected by patients in their ecologic environment, can be used in clinical trials to provide a more realistic view on the target of the clinical investigation (2).

Furthermore, collecting data while the patient is at home and monitoring these data remotely is easier and cheaper for patients compared to the traditional methods that require a constant examination by a medical specialist. Moreover, the possibility to access and monitor data related to disease progression improves patient's awareness and compliance to therapy (3).

However, the increased access to institutional health data (i.e., electronic health records, EHRs), not only for “reading” purposes but also for uploading personally collected data, could result in a loss of control over data quality and reliability, if compared to data acquired by healthcare professionals within the clinical center. Also, patient's personal devices cannot be monitored efficiently and the data stored in them cannot be easily kept safe: malware on personal devices could affect patient' sensitive data or infect the full system; patients could forget to collect some data, even though scheduled, or could lose them for technical issues.

Finally, whether stored on a web platform/cloud system or on the personal device, patient's personal mHealth applications create health-related data sets that, even though potentially relevant for the care pathway, are not integrated with the patient's health record. These data silos form a scattered “island” view requiring to retrieve information from different systems in order to reconstruct the full view on the patient's health pathway (1).

Taken together, these considerations suggest that, despite the potential benefits of personally collected health data for clinical research, their effective use has to be assessed, and solutions or strategies to allow such integration need to be defined.

Data usable in clinical trials have however specific characteristics, knows as “ALCOA” (A–Attributable, L–Legible, C–Contemporaneous, O–Original, A–Accurate) requirements, which were extensively described on FDA guidance on electronic data sources in clinical trials (4). These characteristics are independent from the data source, that can be either an automated system, or an electronic health record, or any other system. “Attributable” means that each data/document, including their modifications or reviews, should be attributed to an author and to a target. This implies that the data collection system needs to keep track of the person/system responsible for the specific information. “Legible” relates to data readability and understandability, implying that a human-readable and human-understandable format has to be provided for visualization. “Contemporaneous” implies that data are synchronously recorded, and that any change on the recording has to be fully tracked: time-stamp, reason for modification, and author's signature are mandatory to obtain reliable data sets. “Original” means that data should be preserved in their original form or in a certified true copy. When considering the mobile environment, and patient-generated data, the concept of “original” assumes and even higher importance because the data collection module needs to accurately record raw data by permanent means at the time of the activity and protect them from any further modifications, in order to preserve originality. “Accurate” regards data thoroughness and honestness for a consistent and real representation of the patient's status, conforming to protocol. For personally-generated data, in which the author is the patient, the concept of “accuracy” should take into account also the problem of reliability due to low patient's health literacy and to the use of personal medical devices that can be not correctly synchronized or maintained. Finally, data collection should follow the clinical investigation protocol, in order to be of enough quality to be used in a clinical investigation.

In order to verify whether personally-collected health data, after being integrated in the care pathway according to the ALCOA requirements, can be used in clinical research, we designed and implemented a standards-based system that uses

- a specifically designed web-based platform that represents the institutional clinical data collection system (5);
- a workflow engine to manage the clinical trial process;
- a set of mobile apps and wearable devices for patient's personal data collection that exchanges information with the web-platform using a standards-based document set.

We tested the system in a clinical investigation for the optimization of deep brain stimulation (DBS) therapy in patients with Parkinson's disease that required the collection of both patient-generated and clinical/neurophysiological data.

## Materials and Methods

### System Architecture

**Figure 1:**
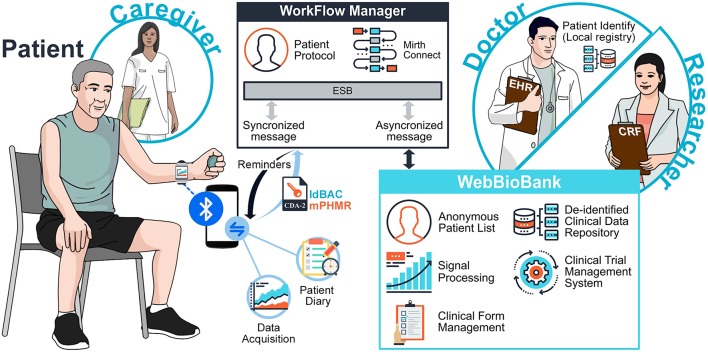
System Architecture.

The system integrates information collected by different actors (Figure 1), namely the Patient, the Doctor, and the Researcher. The Patient may be supported by a Caregiver. The architecture has three main components: The Patient System, the Workflow Manager (WFM), and the WebBioBank (WBB) (5). The system is fully described in Marceglia et al. (6).

In the general process of clinical research, the Researcher defines the measures needed to achieve the research objectives and a data collection process. In the system for data integrated data collection proposed in Figure 1, the Patient collects personal data through the Patient System, while the Doctor collects clinical data through the WBB. The WFM guarantees the correct execution of the research protocol, by implementing the data collection process and workflow (e.g., it reminds the Doctor to assess the patient, or the Patient to switch her/his wearable device on). Thanks to data integration, not only the Doctor can monitor patient's personally collected data, but also the Researchers can access the system and analyze the holistic dataset.

**Figure 2:**
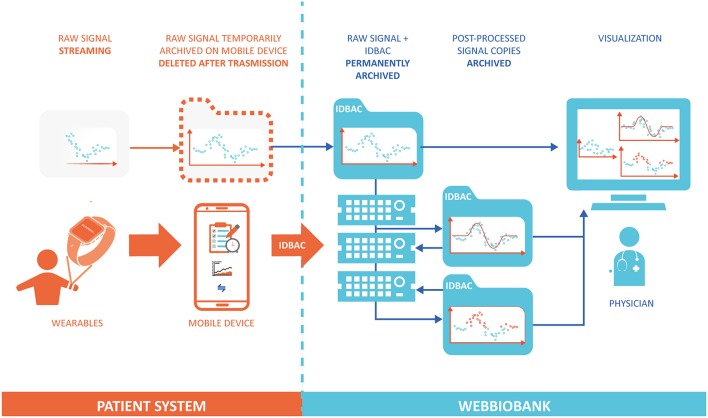
Data flow model between the Patient System and the WebBioBank. The raw data is permanently archived only into the WebBioBank system, and all the post-processed data is created and archived in different copies.

In order to ensure data integrity and respect ALCOA principles, the system provides access only to required and authenticated individuals and implements an audit log in which all date entries, changes, and deletions are mapped, time-stamped, and signed. Before data storing, the data format is automatically inspected to detect any error in data input or caused by data diddling attacks and, to prevent possible loss of data, a backup, and recovery strategy is implemented. Once stored in the Patient repository, the original raw data can be easily accessed via data models with read-only permission. Any automated or manual data manipulation can only use the raw data as input and save the new information in a separate file. Figure 2 shows the workflow of data and signals in the system, from the personal patient's system to the WebBioBank archive.

### System Components

The Patient System is composed by hardware and software, and it includes a Wearable Device (WD), a Mobile Device (MD), and a mHealth app for the Patient and the Caregiver (Figure 1). The WD, in this specific application, is a wearable accelerometer that dialogues with the MD. Patient-reported data (Patient Diary) are collected through the mHealth app and are sent to the WFM via an external gateway.

The exchange of XML-based CDA-2 structured documents between the MD and the WFM ensures anonymous communication (7) and it prevents from the exchange of identification data through unsecure connections, as well as the maintenance of these data into unsafe environments. The CDA-2 template used is a modified version of the Personal Healthcare Monitoring Report (PHMR) CDA2 template (CDAR2_IG_PHMRPTS_R1.1_DSTU_2010OCT) which was changed to fulfill the requirements of the mHealth App/EHR data exchange described in Marceglia et al. (7). The modified template is referred to as mPHMR.

The WFM-Patient System communication is managed through exposed services (e.g., to manage reminders and notifications, to send schedules, etc). The WFM is devoted to protocol definition and management through a graphical interface allowing to design graphical flowcharts in a workflow editor. The Doctor associates the Patient to the specific protocol that is executed in a workflow engine (Mirth Connect health care integration engine) (8).

From WFM, the information is sent to the WBB, a web-based platform for clinical data collection integrated with signal management and processing (5), also for multicenter clinical studies. WBB ensures data de-identification and access control. Anonymous data collection is implemented through the use of unique patients' IDs (IDBAC) instead of any other identifying demographic information. Being unique, the IDBAC ensures attributability of records to patients. WBB is defined as a “research” EHR (rEHR) (5) because it integrates the functionalities of traditional EHRs with those of research support systems.

### Validation Case Study

Telemonitoring of patients with DBS implant is useful since these patients face a fragile stabilization period immediately after electrode placement. We decided to validate this system on these patients after 5 days from the electrode placement in a controlled environment, to keep the risks for patients as low as possible.

**Figure 3:**
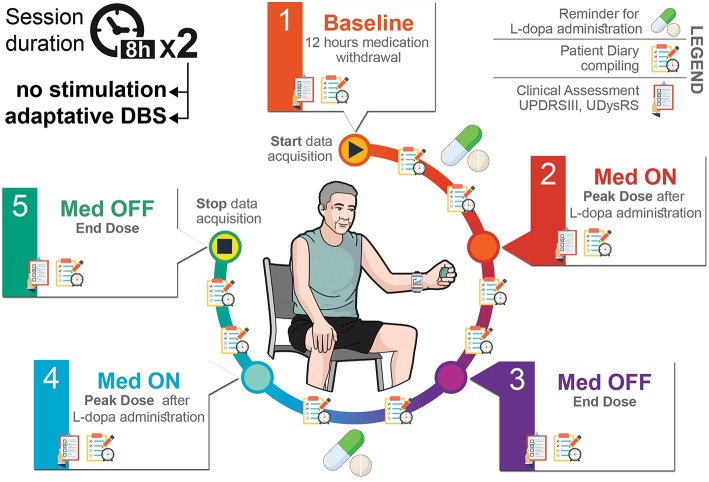
Eight-hours experimental protocol.

The experimental protocol is depicted in Figure 3. This study was carried out in accordance with the recommendations of IRCCS Ca' Granda Ospedale Maggiore Policlinico review board with written informed consent from all subjects, in accordance with the Declaration of Helsinki. The protocol was also approved by and the Italian Ministry of Health. We enrolled 10 PD patients who underwent surgery for DBS electrode implant having externalized leads for a week to connect a wearable device for aDBS testing. Two perioperative sessions lasting from 7 to 8 h were conducted the day 5 and 6 after surgery while the patient was doing his/her normal activities. In the first session, the beta band power was continuously recorded while the patient took its post-operative daily medication dose. In the second session, we added aDBS treatment to beta band power monitoring and daily medication. A neurologist assessed the clinical state and fluctuations at 5 time points (Figure 3) through the motor Unified Parkinson's Disease Rating Scale part III (UPDRSIII) and the Unified Dyskinesia Rating Scale (UDysRS). During the entire duration of the experimental session, the patient filled in a diary (every 30 min) and wore a bracelet to assess his/her bradykinesia state.

### Patient Generated Data Acquisition

Patient generated data consist of personal diary, collecting the patient's state every 30 min with scheduled alerts and a wrist accelerometer acquired by a commercially available wearable device.

Data was collected through a mobile device (Android phones: Motorola X Play and Huawei Nova Young) with a dedicated app that acquires the wrist accelerometer data (a Pebble Time smartwatch with a custom app, connected to the mobile device via Bluetooth) and provides a clinical diary form every 30 min to be filled-in by the patient. The patient has a personal ID and password to access the app.

The mobile phone app implements an algorithm that finds Bradykinesia Acceleration Scores (BAS) using the wrist accelerometric values without specific tests involved, in an ecologic but controlled environment. The BAS algorithm was adapted from the patent of Griffiths and Horne (9).

The app was entirely developed by our team using the native Android Framework connected to the standard Pebble Time Smartwatch app that provides the raw sensor data by the standard intent broadcast.

At the end of the session, as a preliminary feedback on system usability, a clinician asked the patients if the smartwatch was uncomfortable during the day. If the answer was “Yes” the patient was asked to explain why.

### System Testing

The system Validation test consisted in two use-case tests: the first one consists in the interaction of a doctor to fill-in a clinical form on the WBB system, and the second one consists in the collection of patient-generated data through a wearable. The methods and results of the two experiments are reported in Tables 1–4. Tables 1, 3 detail the two primary use case tests of the two experiments and their expected results are reported in Tables 2, 4.

**Table 1:**
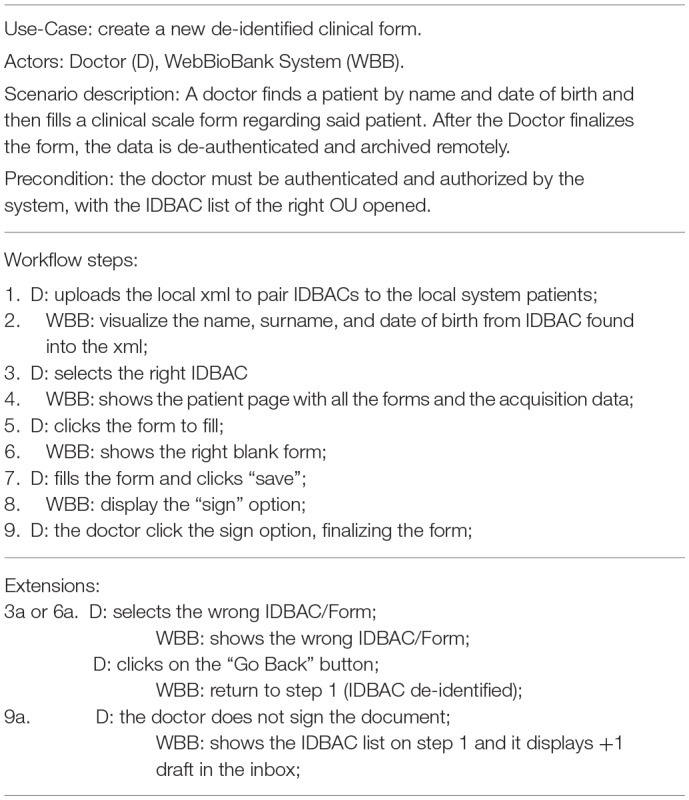
Use Case test for creating a new de-identified clinical form in WBB made by a doctor.

*The Doctor finds the patients in the system and fills the clinical scale form*.

**Table 2:** Use case test results for Table 1.

Expected results: 1. Every System response (WBB) must be carried out correctly. 2. No IDBAC with personal information must remain cached in the grid form except between steps 2 and 3. a. Every personal detail must be only retained on the local database file, no browser must retain this information; b. This data cannot leave the local machine. 3. Signed form cannot be deleted or modified. 4. Saved but not signed forms can be modified.
Results: 1. Every System step was fulfilled, to test this scenario we used UPDRSIII and UDysRS forms; 2a. the system was tested with Google Chrome (ver. 62.0.3202) and Internet Explorer 11 as browsers, no information were visualized on all the other steps except 2,3; 2b. Wireshark (ver. 2.0.3) was used to test the internet/WLAN traffic during this test, no personal data was exchanged outside the local machine; 3. it's not possible to modify or delete the form, also, the “save” and the “sign” operation are logged with the system timestamp (server-side); 4. Saved forms can be modified but not deleted without Administrator's rights.

**Table 3:**
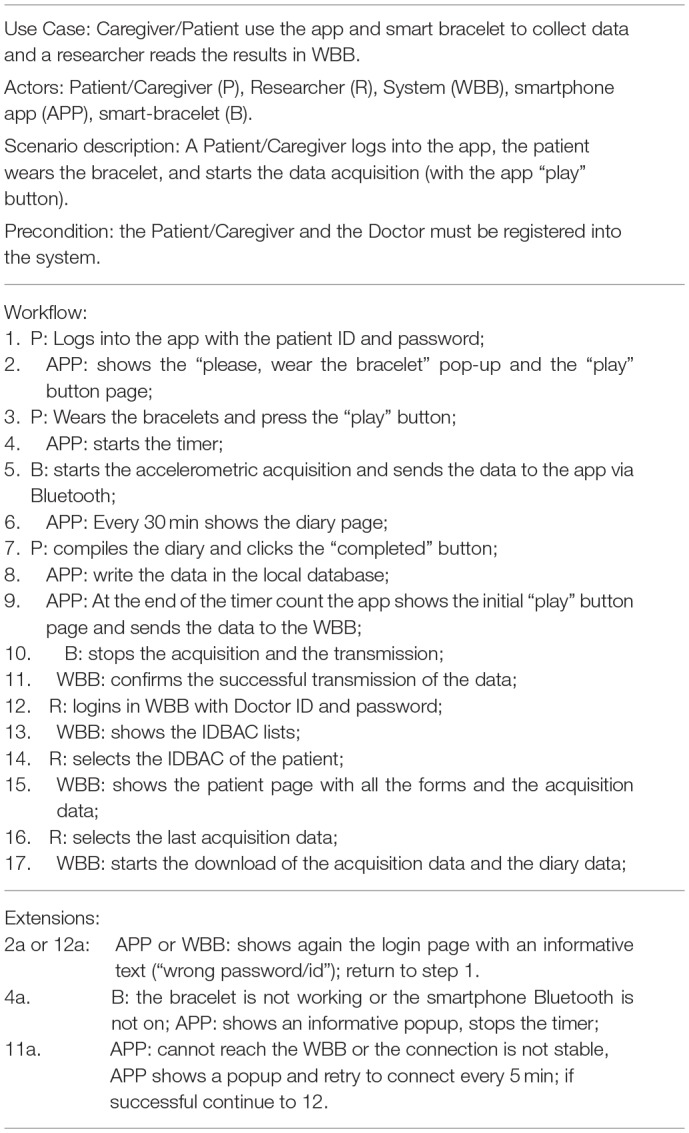
Use Case test for the patient data acquisition.

*The Patient wears the bracelet and starts the data acquisition, logging into the app*.

**Table 4:** Use Case test results for Table 3.

Expected results: 1. Every System response (APP, B, and WBB) must be visualized correctly. 2. Limited data loss during the acquisition process; 3. At least 8 h acquisition session without recharging the smartphone or the bracelet; 4. Downloaded data by the researcher must be in a known readable format (comma separated values or xls, Excel format);
Results: 1. Every System step was fulfilled correctly; 2. Some accelerometric data in particular timeframes were missing due to the distance of the mobile device from the patient caused by forgetfulness or special conditions (e.g., MRI or other exams). In normal operating condition, the data throughput is sufficient to guarantee more than 80 sample/s. 3. Pebble Time and the smartphone (Motorola Moto X Play) fully charged lasted more than 8 h. 4. The data was downloaded and analyzed: The correlation between UPDRSIII scores and the BAS is −0.563, (*p* < 0.0005, Pearson) for 5 sessions regarding 4 different patients.

## Results

### System Implementation

**Figure 4:**
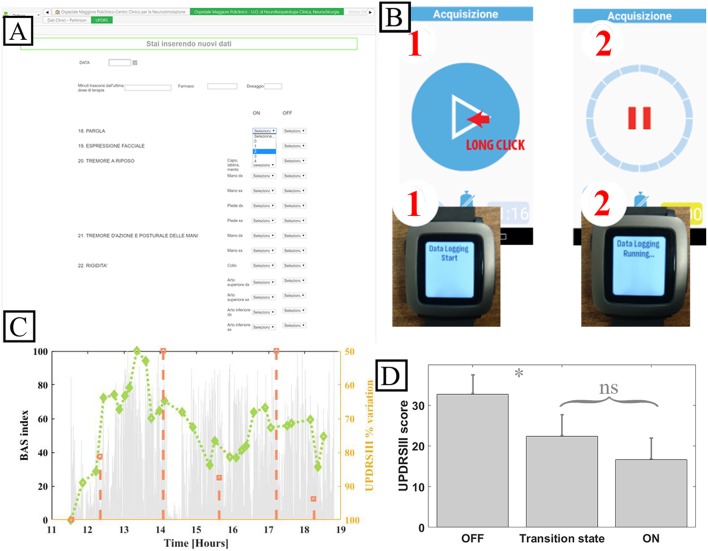
System implementation for the validation case study. **(A)** Snapshot of the WBB form for UPDRS III. **(B)** Patient mobile device and smart bracelet custom app. **(C)** Accelerometer exemplary data. **(D)** Mean UPDRS III scores grouped by patient reported diary state and relative statistical error. ns: not significative. ^*^: *p* < 0.05, Pearson. Note that the system was implemented in Italian.

The system was fully implemented to be used in the validation case study. WBB was configured in terms of users, roles, and forms to support the 8-h research study in the hospital-based ecologic but controlled environment. WBB fulfills the requirements for clinical study data collection: patients are de-identified to ensure security; the clinical forms are developed by the researcher (usually the principal investigator), filled in and signed by the author, reviewed by the principal investigator, and changes/modifications are tracked, time-stamped, and signed whenever they occur, thus guaranteeing integrity, attributability, and reliability. The definition of the “inspector” user role allowed auditability. Figure 4A shows the snapshots of the main rEHR forms created to collect data from PD patients undergoing the experiment.

Two web services were developed to support the communication and integration between the WBB and the WFM. The first one (wHvpc) is devoted to the integration of user roles and patients' IDs: it allows the verification of the privacy criteria for doctors/researchers who access the WFM to create or assign the patient's protocol and, once the doctor accesses to assign the study protocol, allows the exchange of patient's ID and contact information from WBB to WFM. Then, when the protocol has been assigned and the patient is registered in WFM, the WFM deletes contact information and keeps only the patient's ID. In this way, the synchronization between the two systems is guaranteed thus allowing attributability and integrity, and patient's contact information do not reside on WFM, thus allowing security and privacy. The second one (wHcda) is devoted to the exchange of CDA-2 standard documents between the WFM and the WBB. Once the WFM receives patient-generated data from the mobile application, it creates and encrypts the CDA-2 document according to the mPHMR template, and sends it to the WBB using the wHcda web service. Data reliability is therefore ensured by the use of standard documents that are accepted by the WBB platform and processed as clinical documents.

On the patient side, the WFM provides the functionalities for patient's registration and protocol fulfillment. The WFM stores the patient data into the correct rEHR on the WBB by calling the wHcda service passing the identification numbers of the user (uID), the patient (IDBAC), and the custodian (ID OU). The patient data inside the database are anonymous for all users, and only the patient's doctor can re-identify them by means of a local registry. De-identification is guaranteed also by the WFM registration process that does not require patient's demographic information, but uses the contact information retrieved at the time of assignment that are then deleted when the patient is successfully registered. Thanks to the definition of the patient's protocol, the WFM is able to send the activity program to the patient's mobile app, and to provide remainders and alerts when a task is due (e.g., when the patient has to fill-in the diary). In addition, in the case WFM does not receive the patient-generated data on time, according to the protocol, it sends new requests, and then notifies the WBB of the deviation from the protocol, sending a specific CDA-2 with the indication of the deviation using the wHcda web service.

**Figure 5:**
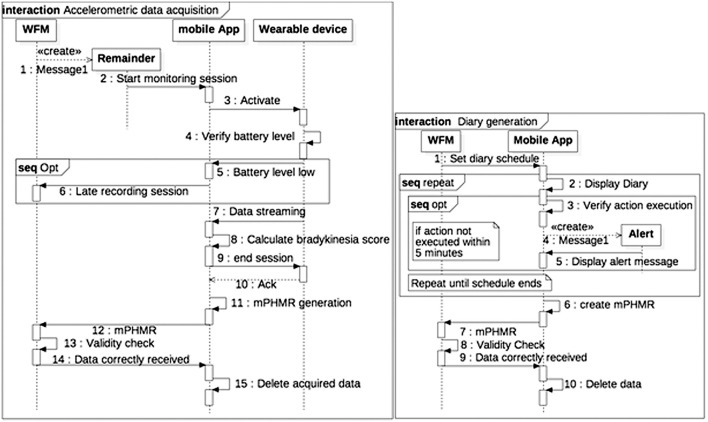
Sequence diagram of Patient-generated data collection. (A) Accelerometric data acquisition. (B) Diary generation.

Patient-generated data collection for the case study, including a patient's diary to be filled in and a wearable bracelet for bradykinesia evaluation (Figure 4B), is shown in the sequence diagrams in Figure 5. The algorithm in the mobile app generates a BAS value every 4 min of data. BAS is lower when the patient is bradykinetic (Figure 4C). Bracelet data are preprocessed in the mobile app to retrieve the bradykinesia score (Figure 4C). The forms for the patient's diary consists of a multiple-choice mutually exclusive list that asks the perceived motor status through 4 different answers: “OFF: Bradykinesia and rigidity,” “ST: Transition,” “ON: normal mobility,” and “ON: disabling dyskinesia.”

The diary and the accelerometer data is stored internally in a SQLite DBMS. When the device is synchronized with the WFM, a mPHMR document is generated with the all the BAS data compressed and encoded in MIME format in an observation of the CDA-2 document (content-type: application/x-compressed, Content-Transfer-Encoding: BASE64) and all the diary data on another plain text observation of the same document. If the mPHMR document is correctly stored and approved by the WFM, a positive feedback is sent back to the mobile device and the internal database is erased for security reason. This feedback and the other remainders are sent through a web-service exposed by the WFM. The use of standard CDA-2 documents between the App and the WFM ensures data integrity, attributability, and safety (in the CDA-2 the author is the patient, identified only by his/her ID). In addition, the mobile app does not retrieve any clinical data, but deletes them when the WFM correctly receives the CDA-2 and validates the data.

### System Validation

In our experiment, we enrolled 10 patients, 2 doctors, and 1 researcher (the principal investigator). In addition, a user with role “inspector” was created to test the audit functionalities. On WBB, the researcher created 7 forms, one for patient's disease history, one for DBS surgery details (target position, electrodes implanted, intraoperative monitoring results), one for the details of the experimental setting (levodopa equivalent dose administered to the patient, neurophysiologic parameters retrieved to set the aDBS device), one for each clinical assessment including the UPDRSIII and the UDysRS scale, and one for the visualization of patient-generated data.

All the expected 140 diary recordings were received by the system. Of them, 117 were filled-in whereas 23 arrived with null values. The major reason was one poor compliant patient who lacked compiling the diary several times. There were no errors in the data transmission. A total of 130 h of accelerometer data were recorded. The accelerometer data loss was due to a poor connection between the wearable device and the mobile app. Despite this, we analyzed 21 paired data points (UPDRSIII scores and the mean BAS scores in a data frame of ± 20 min centered on the UPDRSIII score time) and there was a significant correlation (−0.563, *p* < 0.0005, Pearson); this value is above the 0.5 correlation threshold estimated with a 0.05 alpha error and a power of 70% for 21 data-points (10). This correlation suggests that the measures obtained by the wearable device are reliable for assessment purposes, even though data are incomplete.

We analyzed the first recording session diaries of the four patients correlated with the normalized UPDRSIII scores screened in their relative time frame. A total of 21 paired data (UPDRSIII scores and Diary entries) were assessed. There were no “ON with Dyskinesia” state reported by the patients.

Figure 4D shows the means of the normalized UDPRSIII for all patient's states and the relative statistical error. There are significant differences between “OFF state” and both “transition” and “ON” states (*p* < 0.05, Wilcoxon). Conversely, there was no difference between the transition state and ON state.

The diary categorical answers were also sorted in a ranked order (assigning 4 to the ON Dyskinesia state, 3 to the ON state, 2 to the transition state, and 1 to the OFF state, asleep state were left out). This score correlated well with the UPDRS III scores (−0.7416, *p* < 0.0005, Pearson) and with the accelerometric indexes (0.6042, *p* < 0.05, Pearson).

All patients answered “No” to the smart band tolerance question “was the smartwatch uncomfortable during the day?”, therefore confirming an overall tolerability of the system. The smartphone App tolerance and usability were not assessed not to overburden the recovering patients. The final e-diary data was used to gain a perspective on the compliance. In total, all the expected 140 diary recordings were received by the system. Of them, 117 were filled-in whereas 23 arrived with null values. Of these 23, 20 where filled-in by a single patient.

It is worthy to notice that some of the Caregivers (family members mostly) aided the patients filling-in the eDiary even if instructed not to do that, but it was not possible, with our resources, to monitor this behavior.

## Discussion

In this work, we designed, implemented, and tested the feasibility of a multi-source patient-centered acquisition system in a real clinical research study. Our primary focus was to determine:

- The tolerability of the system to the patient;
- The communication and monitoring infrastructure reliability;
- The quality of the data captured;
- The correlation of these data to a golden standard (UPDRSIII).

This approach follows closely the recommendations steps 4a, 4b, 5, 6 of Clinical Trials Transformation Initiative recommendations (11). In a future study in a truly ecological homecare environment, we plan to conduct usability testing with patients' focus group and a more formal training program. The system integrates personally collected health data into the data acquisition process for a clinical trial, using a standards-based architecture that ensured the fulfillment of the major requirements (4). Our results suggest that the implemented system and architecture were able to provide a reliable solution for including patients as direct digital data source, ensuring ALCOA requirements for data generated either by the patient him/herself of by personal wearable device.

Due to the commercial nature of the personal devices that can be used with this architecture, the low clinical literacy and the lack of clinical supervision may present serious issues on accuracy and reliability. To address these issues, we tested multiple and independent sources (i.e., accelerometric smartband and patient diary) to monitor the correct gathering of these data and give the physician a bigger and more reliable picture related to the same clinical outcome. The accelerometric smartband relies only on wearing and turning on the bracelet/cellphone. Instead, the diary requires a direct self-reporting action by the patient. However, in this feasibility study, we did not test the system usability but only collected data on patient's tolerance (of the smart band) and compliance (through the number of diaries received), not to overburden patients and caregivers. In general, the system was well tolerated, and the compliance was high. However, when fully exploited in the homecare environment, not only a full usability study will be run, but also the design of the App will follow a user-oriented approach, thus limiting intolerability and poor compliance.

The heterogeneity of data given by the use of multiple personal sources can also be used to extract useful information within the interaction of those data, e.g., the patient cognitive status (diaries) vs. his/her motor status (bracelet), or to further understand the issues that patients can have with the device. This approach gives a more patient-centered perspective without overlooking his/her real motor condition.

However, personally generated data do not usually come from app specifically developed and not in controlled environments. These conditions would appear inconsistent with our validation process and highlight the following limitations:

1- the mHealth App was developed *ad hoc*;
2- the validation was performed in a controlled environment;
3- the sample size of patients involved was small.

Regarding the first point, the generalizability across devices and Apps of our system architecture deserves some discussion:

- We used a commercial smartband but not all devices are up to the task, and an appropriate selection must be previously made. The loss of data during the evaluation stage was mainly caused by the low available data storage memory in the bracelet, thus requiring a continuous connection with the mobile phone to transfer data and free the memory. This study shows how the capability of a device to collect data autonomously is critical during the daily living of these fragile patients who are not used to keep the mobile phone close to them but tend to wander off outside its range. This capability will be a major requirement for future implementations of the proposed architecture.
- We used a mHealth App developed *ad hoc*. However, there are no limitations about the Apps linkable to this architecture. The only requirement is the use of standards for both acquisition and exchange of information between the device and the EHR system. This is an essential requirement for make the data relevant from a clinical point of view. The developed App was based on the mPHMR standard but also the latest FHIR standard is fully viable.
- Besides interoperability standards, if a mHealth app performs patient-specific analysis and provides patient-specific diagnosis or treatment recommendations, it must follow a strict quality system regulation indicated in general guidelines (12).
- It has been proposed that interoperability issues in health IT can be addressed by using web services (13, 14). Our architecture, in agreement with this hypothesis, implements specific web services to ensure interoperability among the different systems (WebBioBank, Workflow manager, and mHealth App) and to enable the communication among patients/caregiver, researchers and doctors by using standards such as PHMR template compliant with RIM (CDA2) and dictionaries.

Regarding the controlled environment, we considered that DBS patients after surgery for electrode implant face a fragile stabilization period, and we expected poor compliance and increased risk. For this reason, we decided to make the first assessment of the mHealth App in a controlled hospital environment. However, some characteristics of this controlled environment resembled those of the home environment. In fact, during the experiment, even though patients were without the supervision of the experimenters, they were helped by their families or other informal caregivers in using the system, thus mimicking the home environment. Caregivers (family members mostly) aided the patients filling-in the questionnaire even if instructed not to do that, but it was not possible, with our resources, to monitor this behavior. The main difference between the home and the controlled environments was the initial setup: the experimenters, at the beginning of the day, helped the patients to setup the system, which is of course not possible in the home environment. We therefore anticipate that in a full real-world scenario patients and caregivers will require specific training in order to use the system correctly. For these reasons, the system will require, in the next future, a more focused testing procedure with the patients in their home environment to further verify usability and robustness in longitudinal studies. Finally, further studies of larger populations will be needed to confirm these important finding.

Our results suggest that an integration between patient-generated data and clinical data for supporting research studies is possible. This opens the way for using personally collected health data to improve or facilitate longitudinal research, introducing holistic patient's personal assessment and monitoring.

## Ethics Statement

This study was carried out in accordance with the recommendations of IRCCS CA Granda Ospedale Maggiore Policlinico review board with written informed consent from all subjects. All subjects gave written informed consent in accordance with the Declaration of Helsinki. The protocol was approved by the IRCCS CA Granda Ospedale Maggiore Policlinico review board and the Italian Ministry of Health.

## Author Contributions

PD and SM: design/conception. PD, MP, and RF: literature and database search. PD, MP, LR, AP and SM: experimental protocol design. MP and SB: experiments. PD and MP: data analysis. PD, MP, and SM: writing the initial draft of the manuscript. All authors critically revised and approved the final manuscript.

### Conflict of Interest Statement

LR, RF, SB, AP and SM are stakeholders and founders of Newronika s.r.l., a spin-off company of the Fondazione IRCCS Ca' Granda Ospedale Maggiore Policlinico and Università degli Studi di Milano, Italy. The remaining authors declare that the research was conducted in the absence of any commercial or financial relationships that could be construed as a potential conflict of interest.
